# Electric Cell-Substrate Impedance Sensing (ECIS) as a Convenient Tool to Assess the Potential of Low Molecular Fraction Derived from Medicinal Fungus *Cerrena unicolor* in Action on L929 and CT-26 Cell Lines

**DOI:** 10.3390/molecules27196251

**Published:** 2022-09-22

**Authors:** Monika Prendecka-Wróbel, Dominika Pigoń-Zając, Magdalena Jaszek, Anna Matuszewska, Dawid Stefaniuk, Grzegorz Opielak, Katarzyna Piotrowska, Mansur Rahnama-Hezavah, Teresa Małecka-Massalska

**Affiliations:** 1 Department of Human Physiology, Medical University of Lublin, Radziwiłłowska 11, 20-080 Lublin, Poland; 2 Department of Biochemistry and Biotechnology, Institute of Biological Sciences, Maria Curie-Skłodowska University, Akademicka 19, 20-033 Lublin, Poland; 3 Faculty of Mechanical Engineering, Lublin University of Technology, 20-618 Lublin, Poland; 4 Department of Dental Surgery, Medical University of Lublin, 20-093 Lublin, Poland

**Keywords:** ECIS, impedance, *Cerrena unicolor*, anticancer properties, medicinal fungus, colon cancer

## Abstract

The increase in the incidence of cancer has contributed to the search for new therapeutic methods. In recent years, the use of preparations of natural origin from medical fungi has increased. One such active substance is the extracellular, low molecular active fraction obtained from the medicinal fungus *Cerrena unicolor*. This study aimed to monitor the pharmacokinetics of different concentrations of substances isolated from the medicinal fungus *Cerrena unicolor* (ex-LMS) using the ECIS technique. In the study, mouse L929 fibroblasts and colon cancer CT26 cell lines were treated with different concentrations of the active fractions obtained from *Cerrena unicolor*: C1 = 2.285 (μg/mL); C2 = 22.85 (μg/mL); and C3 = 228.5 (μg/mL). This study demonstrated that the tested preparation from *Cerrena unicolor* had no considerable effect on the resistance, capacitance, and impedance of L929 fibroblast cells, which was an indicator of no significant effect on its physiological processes. At the same time, those parameters exhibited a decrease in colon cancer cell viability. Following our previous and current studies on *Cerrena unicolor*, ex-LMS extracts can be safely used in anticancer therapy or chemoprevention with no significant harmful effects on normal cells.

## 1. Introduction

The progress of medicine is possible thanks to the development of therapies based on the use of new, safer, and more effective therapeutic agents. Research on cell cultures is used in the search for new substances with therapeutic properties. The first step in the search for a new drug is to evaluate the effect of a given compound on cells in in vitro cultures. A modern method used in the study of cell cultures is the analysis of selected electrical parameters of living cells. Cell cultivation on the electrode surface enables the measurement of electrical parameters of the cell membrane, such as capacitance, resistance, and impedance [1]. The analysis of these values allows the assessment of changes in the physiology of the studied culture without the need for cell labeling methods such as fluorescence or radioactive labeling, which kill cells and are necessary for the evaluation of traditional cultures [2]. This innovative and non-invasive method is the ECIS technique (Electric Cell-substrate Impedance Sensing). The ECIS method uses low-voltage alternating current (AC). It flows through the electrodes at the bottom of the culture plate. Changes in cell physiology change the morphology, which results in a change in the impedance of their membranes [2]. Measurements and analyses of changes in electrical impedance enable the recognition of metabolic changes occurring in the cells under study (Applied BioPhysics). The system consists of two electrodes, one smaller working electrode and a larger electrode at the bottom of the culture plate. The electrodes are connected to the recording system. The whole set is placed in an incubator with defined conditions of 37 °C and an atmosphere of 5% CO_2_. The recording system is connected to a computer that reads, processes, and stores the parameters tested. Test cells are inoculated, then sedimented and stuck to the electrode surface. Cell membranes have insulating properties; therefore, their adhesion to the electrode will resist the flowing current. Spaces unoccupied by cells and intercellular connections are places of lower resistance. We will observe changes in the current flow as changes in impedance. It will grow as the cells cover the entire electrode surface, i.e., achieving confluence [1]. The impedance measurement is non-invasive and does not affect the behavior of the cells. Changes in the monitored impedance correspond to the changes in the behavior of the tested cells and are related to their adhesion to the electrode, spreading, proliferation, and reaction to substances present in the environment. Changing the physiology of cells affects their morphology, which can be seen as impedance variations. In addition to impedance, the ECIS system also measures resistance and capacitance, i.e., the capacitive properties of the cell membrane. Changes in electrical parameters are presented as a function of time [1]. The impedance (Z) measured by the ECIS technique is determined by Ohm’s law as the ratio between the current and the voltage in an alternating current circuit. It corresponds to the electrical resistance in DC circuits [3]. The determination of impedance is complicated due to sinusoidal phase shifts between the amplitude and the current in AC circuits. Therefore, in circuits, complex impedance is used, which refers to the amplitude and phase of the current and voltage. Measurements are made every second at specified current frequency values from 1 to 100 kHz. Changes in the frequency value allow us to study various properties of the cells under study. This is due to the difference in AC flow at different frequencies. At frequencies below 2 kHz, most of the current flows through the intercellular spaces and the surrounding solution. At frequencies above 4 kHz, most of the current flows through cell membranes. The knowledge of these relationships in research allows for the assessment of the barrier function of cells, their permeability, and membrane capacitance. In pharmaceutical research on new preparations, the ECIS technique plays a key role in determining cytotoxicity. Its use enables the detection of harmful components in pharmaceuticals that lead to cell damage or death.

Among many species, the genus *Cerrena* seems to be the most interesting, mainly due to its wide therapeutic effect [4,5]. According to the available research, biologically active substances obtained from *C. unicolor* can be used in cancer treatment.

Jaszek et al. [5] reported that low molecular subfraction (ex-LMS) from *Cerrena unicolor* has a strong reducing effect and high ROS-scavenging potential. They concluded that ex-LMS may be potentially used as an effective antioxidant that can be easily produced in controlled laboratory conditions. The ex-LMS sample also showed antibacterial activity against both *E. coli* and *S. aureus* [5]. Therefore, the potential anticancer properties of this preparation seem interesting. In our study, we present a useful and effective method of assessing the impact of ex-LMS on the cells and their properties.

## 2. Results

### 2.1. The MTT Results

The MTT viability test was performed on selected cell lines during a 48-h study. The IC_50_ ex-LMS values for both cell types are listed in Table 1. As can be seen, the preparation showed an antiproliferative effect against CT-26 tumor cells at an IC_50_ value of 23.6 µg/mL, which is much stronger than against L929 cells (IC50 value of 347.5 µg/mL).

### 2.2. The ECIS Results

The research hypothesis of the presented study was based on literature reports indicating possible antiproliferative and proapoptotic activity of fungal preparations ex-LMS and the probability of showing the character and dynamics of these changes using selected electrical parameters, i.e., impedance, resistance, and cell membrane capacity. The obtained results showed that different doses of ex-LMS affect electrical parameters in different modes. Nevertheless, every cell type has its own adhesion characteristics and growth curves that can be manipulated by, e.g., varying seeding density or other stimuli.

**Figure 1:**
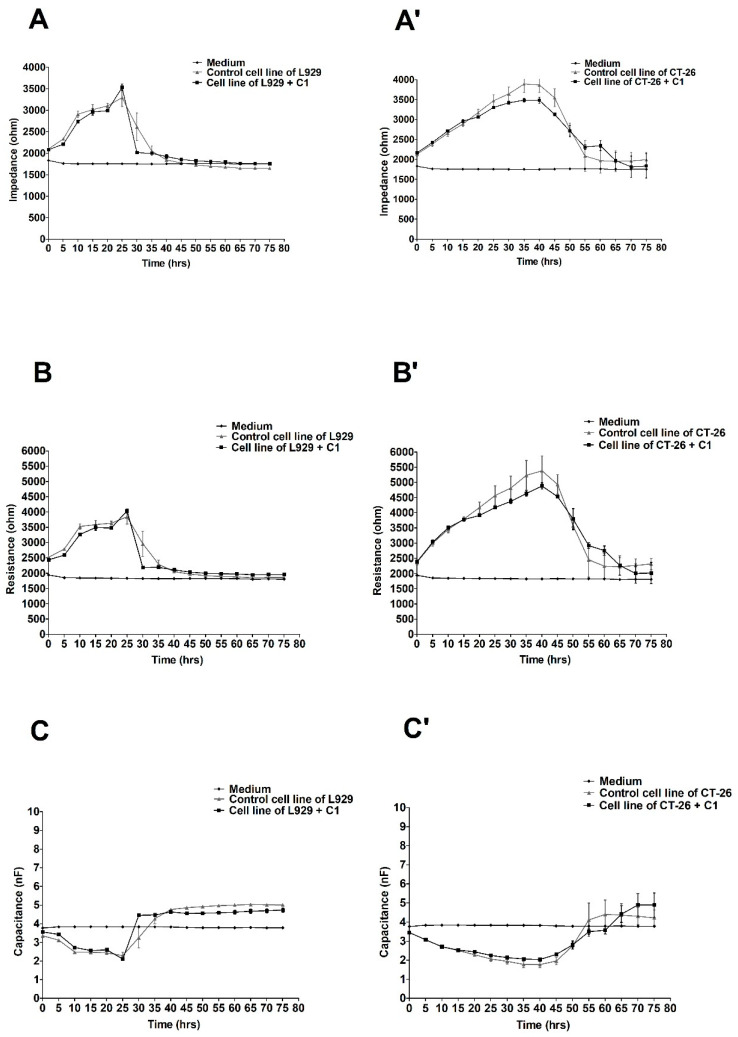
Impedance, resistance, and capacitance monitoring of the cell lines L929 (**A**–**C**) and CT-26 (**A’**–**C’**) during 80 h treatment with ex-LMS in concentration C1. The graphs show the course of impedance changes in L929 (**A**) and CT-26 (**A’**) cultures after treatment with ex-LMS at a concentration of C1 (2.28 µg/mL); resistance changes in L929 (**B**) and CT-26 (**B’**) cultures after treatment with ex-LMS at a concentration of C1 (2.28 µg/mL); and capacitance changes in L929 (**C**) and CT-26 (**C’**) cultures after treatment with ex-LMS at a concentration of C1 (2.28 µg/mL). Data are presented as mean value ± SEM.

The effect of varying ex-LMS concentrations on L929 and CT-26 monolayer and electric changes were continuously monitored for up to 80 h. Using the ECIS system, we recorded the important changes in impedance, resistance, and capacitance in CT-26 cells, but they were only insignificant in L929 cells following the administration of three different concentrations of ex-LMS preparation. Figure 1 shows the changes in impedance, resistance, and capacitance in cultures of L929 (A–C) and CT-26 (A’–C’) cells treated with the preparation at the concentration of C1 (2.28 µg/mL). As can be seen, the lowest concentration of ex-LMS only slightly decreased the viability of colorectal cancer cells (A’–C’). The impedance values in this variant for the cells treated with the preparation decreased to only 3500 ohms between 30 and 40 h of operation, while at the same time, the control cells showed relatively the best viability expressed in impedance of about 4000 ohms. Fibroblast cells behaved slightly differently under the same conditions and after treatment with the same dose of the preparation (Figure 1A–C). There was a clear decrease in impedance in the L929 culture after 30 h of the experiment. As can be seen, control cells and cells treated with the preparation (C1) showed almost identical viability. The peak impedance value was obtained at 25 h (approximately 3500 ohms). The resistance also remained at a similar level, and the greatest drop in capacitance was recorded at the 25th hour of the experiment.

**Figure 2:**
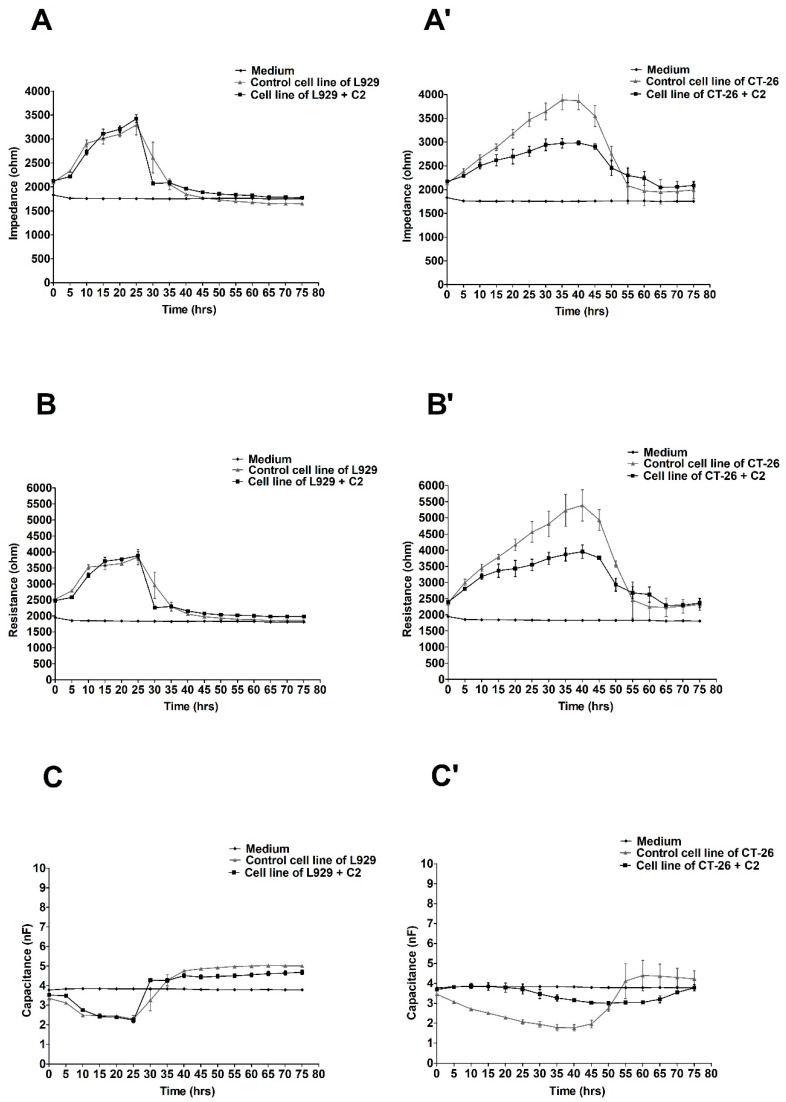
Impedance, resistance, and capacitance monitoring of the cell lines L929 (**A**–**C**) and CT-26 (**A’**–**C’**) during 80 h treatment with ex-LMS in concentration C2. The graphs show the course of impedance changes in L929 (**A**) and CT-26 (**A’**) cultures after treatment with ex-LMS at a concentration of C2 (22.85 µg/mL); resistance changes in L929 (**B**) and CT-26 (**B’**) cultures after treatment with ex-LMS at a concentration of C2 (22.85 µg/mL); and capacitance changes in L929 (**C**) and CT-26 (**C’**) cultures after treatment with ex-LMS at a concentration of C2 (22.85 µg/mL). Data are presented as mean value ± SEM.

Figure 2 shows the changes in the values of electrical parameters in cultures L929 (Figure 2A–C) and CT-26 (Figure 2A’–C’) after the application of ex-LMS at a concentration of C2 (22.85 µg/mL).

After administration of the concentration of 22.85 µg/mL (C2) to the cells, there was a stronger effect of ex-LMS on tumor cells than in the case of the concentration of C1. Adequate changes in all electrical parameters were found after applying the C2 ex-LMS concentration in the culture of CT-26. There was a decrease in impedance in the CT-26 culture to a value below 3000 ohms, while at the same time, cells not treated with the preparation reached an impedance of about 4000 ohms (Figure 2A’–C’). At the same time, the study was performed for the variant with normal L929 cells. In this case, there were no significant differences in the values of impedance, resistance, and capacitance after administering ex-LMS at the C2 concentration.

**Figure 3:**
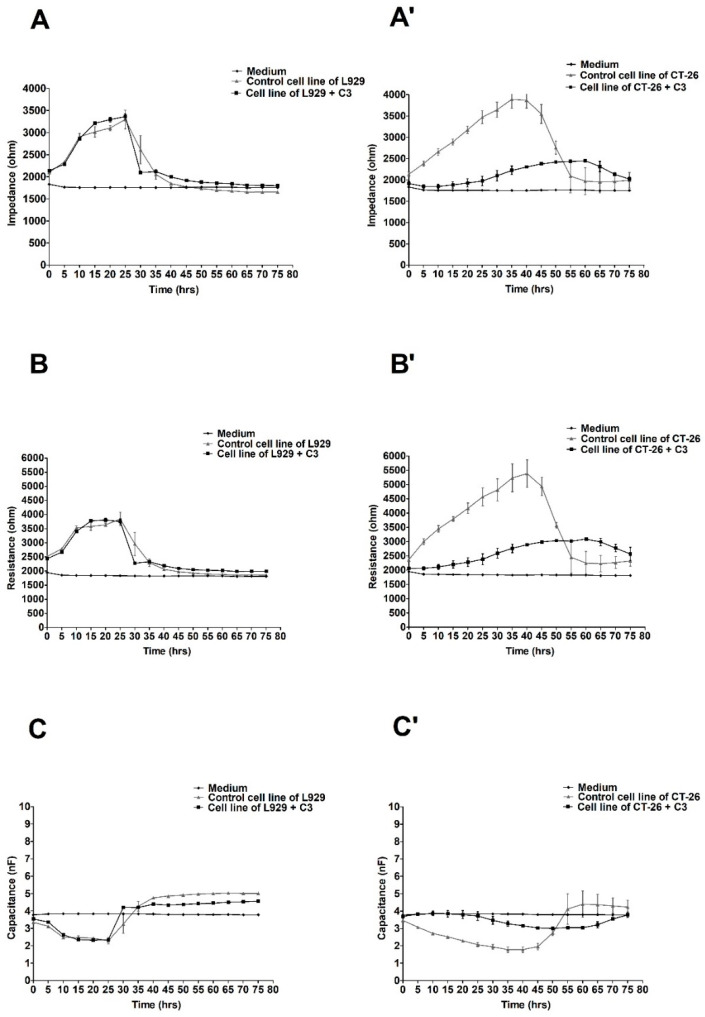
Impedance, resistance, and capacitance monitoring of the cell lines L929 (**A**–**C**) and CT-26 (**A’**–**C’**) during 80 h treatment with ex-LMS in concentration C3. The graphs show the course of impedance changes in L929 (**A**) and CT-26 (**A’**) cultures after treatment with ex-LMS at a concentration of C3 (228.5 µg/mL); resistance changes in L929 (**B**) and CT-26 (**B’**) cultures after treatment with ex-LMS at a concentration of C3 (228.5 µg/mL); and capacitance changes in L929 (**C**) and CT-26 (**C’**) cultures after treatment with ex-LMS at a concentration of C3 (228.5 µg/mL). Data are presented as mean value ± SEM.

The most significant differences in electrical parameters in both cultures (L929 and CT-26) were noted after treating the cells of both lines with the preparation at the concentration of C3 (228.5 mg/mL). The above values are illustrated in Figure 3.

In this case, the values of impedance and resistance in the culture of CT-26 treated with ex-LMS at the concentration of C3 significantly differed from the values of the above parameters for the tumor cells not treated with the preparation. There was a decrease in impedance and resistance to the level of about 2000 ohms, while in the control culture, it remained at the level of an even 4000 ohms. In addition, the capacitance values confirm the trend (for cells treated with the preparation) as being close to the capacitance value of the culture medium. Moreover, once again, no significant effect of the tested substance on fibroblast cells was noticed, which seems to be a positive effect (Figure 3A–C).

**Figure 4:**
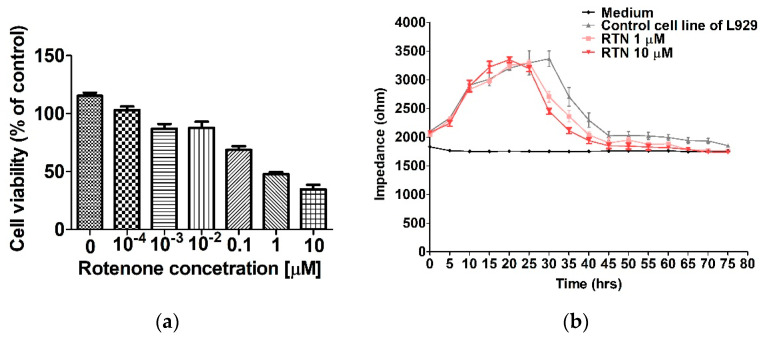
Influence of rotenone on the L929 cell line assessed by (**a**) the MTT assay and monitored by (**b**) the ECIS method. Data are presented as mean value ± SEM.

Additionally, rotenone was used to demonstrate the effect of a known cytotoxic substance on normal L929 cells. Cell viability was assessed in the MTT assay, lasting 48 h (Figure 4a). The next step was selecting two rotenone concentrations and monitoring their performance by ECIS (Figure 4b). As can be seen, both applied rotenone concentrations had a slight effect on cell viability up to the 25th hour of the experiment and then caused a decrease in impedance of more than 1000 ohms at both concentrations after 30 h. The control lines showed better viability as the impedance values did not start to drop noticeably until after the 35-h test but were still higher than in cells treated with rotenone.

## 3. Discussion

One of the most frequently studied biological properties of fungal extracts is their antitumor activity. The antitumor effect depends on their immunomodulatory activities, which are affected by many physical and chemical properties [6,7,8]. It was previously described by Jaszek et al., Matuszewska et al., and Pięt et al. [5,9,10] that there are many new ways and possibilities of using the bioactive waste products formed during metabolic processes by *C. unicolor*. Those compounds may be a source of, for example, low molecular antioxidants, simple sugars, or active peptides. The results showed that *C. unicolor* extracts exhibited antioxidative, antitumor, toxic, and antibacterial effects. Matuszewska et al. demonstrated that laccase essence (ex-LAC) purified from *C. unicolor* exhibited cytotoxic activity against several hematological malignancies and primary CLL cells using an XTT assay, and those findings were confirmed by an apoptosis analysis using flow cytometry and additionally visualized under SEM and fluorescence microscopy [11]. This correlates with our results, where the ECIS technique was used. We have checked the potential anticancer effect of *C. unicolor* extracts on CT-26 cells.

Intensive research on the method’s development is the reason for the attempts to explain the relationship between electrical changes in cells or on their surface and the processes influencing survival, which require a holistic approach, i.e., the application of traditional molecular methods, thanks to which the examination of protein expression (pro and antiapoptotic) and their coding genes lets us obtain information on how bioactive substances influence processes conditioning survival (including apoptosis). No matter how satisfactory this answer might be, in the face of new technical possibilities (ECIS) and if changes in the electrical properties of cells precede changes on the biochemical level, it would be very interesting to examine the character and dynamics of these changes. This is only possible with the monitoring of selected electrical parameters, i.e., impedance, resistance, and capacity of a cell membrane in real time after the application of chosen bioactive compounds to the examined cell lines, which was the aim of this study [12,13].

Drug discovery and screening of bioactive compounds are often performed in cell-based test systems to reduce costs and save time. Traditionally, microscopy, spectrophotometry, colorimetry, ELISA, and flow cytometry techniques are used to examine cell cultures. The above-mentioned methods are considered standard in studies conducted on cell cultures. While these methods may provide insight into the physiological function of every single cell or into pathological changes that could have occurred, they usually require fluorescence, chemiluminescence, or radioactive ways of marking, which may lead to cell destruction [1,14]. The marking process causes the loss of important biological information about living cells. The dead cells are easily detached from the electrode surface. The changes in cell adherence to the electrode surface result in changes in ECIS measurement data. Therefore, cell adherence can be reflected by ECIS data [15]. When performing impedance measurements on intact cells, due to the characteristics of their membrane, cells act as a parallel connection of resistor and capacitor. Here, resistance represents the opposition to current flow, whereas capacitance (C) describes the separation of electric carriers at the insulating bilayer of the cell membrane that causes polarization of the cell. Capacitance provides an overall measure of electrode coverage [16].

Therefore, the different behavior of the cells after the seeding, adherence, proliferation, and their reaction to substances added to the substrate will, as a result, produce a change in impedance. Changes in monitored impedance correspond to changes in the behavior of the tested cells and are related to their adhesion to the electrode, spreading, proliferation, and reaction to substances present in the environment. Cell membranes have insulating properties; therefore, their adhesion to the electrode will resist the flowing current. Spaces unoccupied by cells and intercellular connections are places of lower resistance. We will observe changes in the current flow as changes in impedance. It will grow as the cells cover the entire electrode surface [1].

From the measurement of capacitance, it can be concluded that higher frequencies are most suited for cell spreading because they increase the cell coverage of the electrode. At high-frequency ranges, measurement is more sensitive to changes in cell attachment and spreading at the electrode [1]. Nevertheless, according to Wegener et al., 10–40 kHz is the main frequency range used for measuring impedance [2]. The results of our work remain in compliance with the studies of Hofman et al., which found that impedance readings at 32 kHz are the most robust indicator for cytotoxicity [17]. Electrical impedance is defined as the opposition to the electrical current within a circuit. In systems utilizing direct current, the impedance is simply the resistance, but in systems utilizing alternating currents, the changing electric and magnetic fields create additional and varying opposition to the applied current [18].

## 4. Materials and Methods

### 4.1. Microorganism and Culture Conditions

*C. unicolor* (Bull. ex Fr. Murr.) was obtained from the culture collection of the Regensburg University and deposited in the fungal collection of the Department of Biochemistry (Maria Curie-Skłodowska University, Poland) under strain number 139 (ITS sequence deposited in GenBank under accession number DQ056858) [19].

The *C. unicolor* fermentor scale cultivation was performed in a 2.5 L Bioflo III (New Brunswick Scientific, New Brunswick, NJ, USA) fermentor containing 2 L of a sterilized Lindenberg and Holm medium optimized at 28 °C as in [20]. The fermentor was inoculated (10% of total volume), aerated at 1 L of air per minute, and stirred at 100 rpm. Antifoam B emulsion (Sigma, St. Louis, MO, USA) was occasionally added to the fermentor cultures to break the foam. The determination of the idiophase beginning was performed as in [21]. Subsequently, 10-day-old idiophasic cultures were filtered through Miracloth (Calbiochem) and used for further assays.

### 4.2. Preparation of the Extracellular Low Molecular Weight Subfractions (ex-LMS)

The separation of the culture fluid subfraction below 10 kDa was performed via ultrafiltration with Biomax 10 membrane (10 kDa cut-off; Merck, Darmstadt, Germany) and was concentrated using a reverse osmosis column. It was lyophilized and used as a source of natural low molecular weight metabolites (extracellular low molecular weight subfraction, ex-LMS). The samples of ex-LMS were dissolved in distilled water at a concentration of 1 mg/mL and used for further analyses. The amounts of total and reducing carbohydrates, proteins, and phenolic compounds were estimated in an earlier report [5].

### 4.3. Analytical Methods—Determination of Carbohydrates, Proteins, and Phenolic Compounds

The concentration of the reducing sugars was measured using the Somogyi–Nelson method [22]. The total carbohydrate content of ex-LMS was determined according to Dubois et al. using the phenol-sulfuric acid assay with D-glucose as a standard [23]. The protein concentration in the ex-LMS was determined using Bradford reagent and bovine serum albumin as a standard [24]. The total phenolic compound content was determined with diazosulfanilamide using the DASA test, where the absorbance was measured at 500 nm and using vanillic acid as the standard [25].

### 4.4. FT-IR Spectroscopy Analysis of ex-LMS Sample

The analyses of ex-LMS were performed using the crude, lyophilized filtrate obtained from the concentrated culture fluid separated with a Biomax 10 kDa membrane. FTIR spectroscopy was recorded with a spectrometer (Thermo Scientific Nicolet 8700A with FT Ramana Nicolet NXR module, Waltham, MA, USA) in the wavelength range of 4000–400 cm^−1^.

The number of proteins, total polysaccharides, reducing sugar, and total phenolic compound content of ex-LMS were estimated in an earlier report [5] (Table 2).

### 4.5. Cell Lines and Culture Conditions

In the initial phase, cells of mouse fibroblast cell line—NCTC clone 929 (L cell, L-929, derivative of Strain L)(ATCC^®^ CCL-1™) and a culture of colon carcinoma CT-26 (*N*-nitroso-*N*-methylurethane-(NNMU)-induced, undifferentiated colon carcinoma cell line, cloned to generate the cell line designated CT26.WT, ATCC^®^ CRL-2638™) were derived from ATCC and cultured according to the manual [26,27].

The examined cell lines, L-929 and CT26, were cultured in complete MEM Eagle (Pan-Biotech) and RPMI-1640 (Sigma-Aldrich, St. Louis, MO, USA), respectively, supplemented with 10% FBS Good HI (Sigma-Aldrich) and antibiotics (100 IU/mL penicillin, 10 mg/mL streptomycin, and 25 µg/mL amphotericin B, Pan-Biotech, Aidenbach, Germany) in a Galaxy 170R incubator under controlled growth conditions, constant humidity, and air saturation of 5% CO_2_. After multiplication and stabilization of the cells (approx. 7–14 days), when the culture reached at least 75% confluence, the next stage of the examination was culturing the cells with ex-LMS in different concentrations.

### 4.6. The Cell Proliferation Assay—MTT Assay

The MTT assay, involving 3-(4,5-dimethylthiazol-2-yl)-2,5-diphenyltetrazolium bromide (Roche, Basel, Switzerland), was used to examine the cell viability. In this test, the yellow tetrazolium salt was metabolized by viable cells into purple formazan crystals. L929 and CT-26 cells were seeded on 96-well microplate (Nunc) at a density of 1 × 10^4^ cells/well and left for 24 h. The next day, the culture medium was removed, and the cells were exposed to serial dilutions (0.2285, 2.285, 22.85, 228.5, and 350 µg/mL of ex-LMS made in a serum-free medium for 48 h). Extreme concentrations were discarded later in the study. Moreover, the L929 cell line was exposed to rotenone at the following concentrations: 0.1, 1, 10, 100 nM, 1, and 10 μM. The examined compound in each concentration was tested in triplicate. The next step was the incubation of cells for 4 h with 20 μL of MTT solution (5 mg/mL). The formazan grains formed by viable cells were solubilized with 200 μL of DMSO, and the color intensity was measured at a 570 nm wavelength. As we previously described, the results were expressed as an IC_50_—the concentration of compound (in μM) that inhibits the proliferation rate of the tumor cells by 50% when compared to the untreated control cells [28]. The experiment was performed with three independent repetitions.

### 4.7. The Electric Cell-Substrate Impedance Sensing

The ECIS system (Applied Biophysics, Inc. Troy, NY, USA) was used to measure impedance, resistance, and capacitance. It contained two separate units: the station controller Zθ, located outside the incubator, and a docking station containing the station (96 wells) placed within the incubator space. The standard, 96-well, disposable ECIS arrays are made of gold electrodes insulated with a special insulating foil and installed on glass fiber supports. The 96-well plate is made of polystyrene, and each culture well is about 0.3 cm^2^ and holds a maximum volume of around 400 microliters. In this type of plate (96W1E), each of the 96 wells has one active measuring electrode with a diameter of 250 μm [29]. ECIS electrodes are placed in a holder plate in a humid incubator (Galaxy 170R) at 37 °C and 5% CO_2_. Before inoculation, the arrays were incubated for 24 h with Eagle MEM (L-929) and RPMI (CT26) in the Galaxy 170R incubator overnight. Following stabilization, the array was removed from the array station and inoculated with cells.

Inoculation of arrays was carried out with 600 microliters per well of cell suspension of about 1.2 × 105 cells/mL. Ex-LMS was added to inoculated wells of the final concentrations: 2.285 μg/mL (C1); 22.85 μg/mL (C2); and 228.5 μg/mL (C3). After cell manipulation, the matrix holder was placed in an incubator, and real-time measurements were initiated. The maximum response for Z, R, and C occurred at different frequencies. In this study, the default optimal frequencies were used: resistance (R) 4000 Hz, impedance (Z) 32,000 Hz, and capacitance (C) 64,000 Hz.

## 5. Conclusions

The results of our research confirm the thesis that the fungal preparations ex-LMS showed antiproliferative and proapoptotic activity against the CT-26 cell line. This is indicated by a significant decrease in impedance and resistance parameters in the CT-26 cell line after being treated with different concentrations of ex-LMS compared to untreated cells and normal cells. In the case of colon cancer cells, a drop in impedance and resistance suggests a decrease in cell viability, while not influencing the physiological activities of L929 fibroblasts. This may indicate the possibility of using the tested preparation in anticancer therapy. In the present study, we followed the dynamics of biologically active substance interactions with the CT-26 cell line. These data, in combination with more classical techniques, such as ELISA or Western blot analyses, will certainly allow an understanding of the mechanisms influencing the activation of apoptotic processes in neoplastic cells in the future.

## Figures and Tables

**Table 1:** IC_50_ values of ex-LMS on CT-26 and L929 cell lines as determined during 48 h period of MTT assay, using non-linear, four-parameter regression analysis.

Cell Line	IC_50_ [µg/mL]
CT-26	23.6
L929	347.5

**Table 2:** Number of proteins, total polysaccharides, reducing sugar, and total phenolic compound content of tested ex-LMS preparation [5].

Preparation	Protein (µg/mL)	Total Carbohydrate (µg/mL)	Total Polysaccharides (µg/mL)	Reducin Sugars (µg/mL)	Total Phenolic (µM)
Ex-LMS	188.97 ± 1.3	780.07 ± 2.7	272.93 ± 2.7	507.14 ± 2.8	15.0 ± 0.4

## Data Availability

Not applicable.

## Sample Availability

Samples of the compounds are not available from the authors.
